# The menace of self citation: An audit of two years from journals of Khyber Pakhtunkhwa

**DOI:** 10.12669/pjms.38.8.6571

**Published:** 2022

**Authors:** Mian Saad Ahmed, Umema Zafar, Hamna Zafar, Maryam Shahid Ullah, Feroz Ali Khan

**Affiliations:** 1 Dr. Mian Saad Ahmed, MBBS, DPH, DMJ, MPH, MSHCM Department of Forensic Medicine, Khyber Medical College, Peshawar, Pakistan; 2 Dr. Umema Zafar, MBBS, MPhil Department of Physiology, Khyber Medical College, Peshawar, Pakistan; 3 Dr. Hamna Zafar, MBBS Department of Medicine, Khyber Medical College, Peshawar, Pakistan; 4 Maryam Shahid Ullah, MBBS Department of Physiology, Khyber Medical College, Peshawar, Pakistan; 5 Feroz Ali Khan, MBBS Child and Adolescent mental health services, NHS Foundation Trust, Bradford District Care, UK

**Keywords:** Author self-citation, Journal self-citation, Home authorship, Audit

## Abstract

**Objective:**

To assess the author and journal self-citation amongst journals of Khyber Pakhtunkhwa.

**Methods:**

This is a cross-sectional study conducted from January 2021 to July 2021. In total, manuscript published in 10 journals of Khyber Pakhtunkhwa, either recognized by the Higher Education Commission or Pakistan Medical Commission, in the years 2018 and 2019 were included in the present research. All types of manuscripts were analyzed using a pre-designed data extraction table. Results were extracted, analyzed and appropriate statistics were applied.

**Results:**

About 1235 manuscripts published in 68 issues over a period of two years’ time were analyzed. The majority of manuscripts were 1039 (84.1%) original articles followed by case reports 90 (7.3%). Author self-citation came out to be 11.26% and journal self-citation was 6.5%. The same institute’s author affiliation came out to be 40.6%.

**Conclusion:**

The trend of author self-citation was found to be high while that of journal self-citation was low when compared with already prevalent literature.

## INTRODUCTION

The convention of citing one’s previous research in a new publication is author self-citation. It differs from journal self-citation in which previous research is cited in a, to be published manuscript, in the same journal.^1^,^2^The influence of self-citation whether positive or negative depends on its intent. It can be taken as a positive practice if it’s used to refer readers to a previously published useful research or if it is used, in case of lack of literature on a particular topic. The scale tips to the negative side once it’s used for personal gain; to increase the citation score of oneself (authors and journals both).^3^

There can be various factors related to self-citation. The need may arise in case of a novel or rare topic. High self-citation (both authors and journals) can be the increased number of self-citations in comparison to the total number of citations received. Another factor can be information prejudice; the authors/readers cite the journals they are most aware of.^4^ Another possible reason for this practice could be the journal policies or the lack of the same. Elsevier has adopted the following policy “an editor should never conduct any practice that obliges authors to cite his or her journal either as an implied or explicit condition of acceptance for publication… Editors should direct authors to relevant literature as part of the peer-review process; however, this should never extend to blanket instructions to cite individual journals”.^5^

Jahani classifies author self-citation into two categories: Synchronous and diachronous. The former is the percentage ratio of self-citation by an author for a particular publication to all the references included in the same publication. For example, if an article has 10 citations out of which 4 are from the author’s own research, then synchronous self-citation would be 4/10 or 40%. The diachronous self-citation is the ratio of the author’s self-citation of that particular paper in other researches, to the total citations received by that publication in toto. For example, if one of 4 citations that a paper receives belongs to its author, the diachronous self-citation would be 1/4 or 25% (6). The present research focuses on the synchronous type of self-citation.

Dueñas used the term self-reference and pointed out its merits. According to him it augments the research stance of the author, refereeing to one’s own research makes the author seem like a well involved and worthy academician and also solidifies the author’s claims (7). It’s important to comprehend why there’s a link between self-citations and citations from others. Fowler and Aksnes (2007) investigated the role of self-citations in attracting international citations. They conducted a macro-level analysis using a dataset from the Science Citation Index that included citations to Norwegian scientists’ articles. Their findings were fascinating, demonstrating that the more one author mentions his or her own work, the more it gets cited by other experts. As the number of citations, co-authorship, and publications increased, the likelihood of self-citation increased as well. After one year, each supplemental self-citation increases the number of citations from others by about one citation, and after five years, it increases by nearly three citations.^8^

A publication in PLoS Biology showed that out of 100,000 top researchers 250 scientists had accrued more than 50% of their citations from themselves or their co-authors, whereas the average self-citation rate was 12.7%.^9^ In the year 2018 Pakistan Medical and Dental Council introduced a policy for promotion of faculty of Medical/Dental institutes which was to be implemented from 2019. According to it first six authors got equal credit for a publication. This necessitated an analysis of the publications, pre, and post, the policy implementation, to analyze its impact on publication practices. The aim of this research was to evaluate the frequency of self-citation by an author or a journal among the Medical Journals of KPK.

## METHODS

This cross-sectional study was conducted on ten journals of Khyber Pakhtunkhwa Pakistan for manuscripts published in 2018 and 2019 from January 2021 to July 2021. Ethics Committee approval was obtained with No.1010/DME/KMC and dated January 12^th^, 2021. The study opted a proper journal inclusion criterion, wherein Higher Education Commission or, Pakistan Medical Commission (Erstwhile PMDC) recognized and open access journals with all its issues published in 2018 and 2019 were enrolled in this study. In terms of manuscript selection, all the manuscripts published in the ten journals of Khyber Pakhtunkhwa were included; while manuscripts with incomplete information and corrupt webpage leading to failure of online download were excluded from this study. A pre-designed proforma was used to extract the information. The extracted information was converted into datasets using SPSS v.25.0. Descriptive statistics like frequencies were calculated for categorical variables; while numerical variables were presented in the form of averages.

## RESULTS

The study in ten journals of Khyber Pakhtunkhwa Pakistan analyzed 1,235 manuscripts published in 68 issues for the two years’ time period (2018 to 2019) with an average of 61.75 articles per year per journal and 18.16 articles per issue. As per details of articles published in different journals, Journal of Ayub Medical College, Abbottabad has published maximum manuscripts 319 (25.8%), followed by 202 (16.4%), rest the details of articles published in different journals is given in Table-I.

**Table-I:** Articles published in different journals.

Name of the Journal	Articles published N (%)
Journal of Ayub Medical College, Abbotabad	319(25.8)
Khyber Journal of Medical Sciences, Peshawar	202(16.4)
Journal of Medical Science, Peshawar	144(11.7)
Journal of Postgraduate Medical Institute, Peshawar	139(11.3)
Journal of Khyber College of Dentistry, Peshawar	122(9.9)
Journal of Saidu Medical College, Swat	110(8.9)
Khyber Medical University Journal, Kohat	77(6.2)
Gomal Journal of Medical Sciences, DI Khan	60(4.9)
Advances in Basic Medical Sciences, Peshawar	36 (2.9)
Journal of Gandhara Medical and Dental Science, Peshawar	26(2.1)

***Number of references per articles***	***N (%)***

0-30	1089(88.2)
31-60	137(11.1)
61-100	8(0.6)
101-120	1(0.1)

***Number of authors per articles***	***N (%***)

1-6	1118 (90.5)
7-14	115 (9.3)
15-20	2(0.2)

In year wise distribution, the data shows that 615 (49.8%) articles were published in 2018, and 620 (50.2%) articles were published in 2019. The journals of Khyber Pakhtunkhwa published 12 (twelve) different types of manuscript in these two years, wherein after Original Article 1,039 (84.1%), Case Reports were published the most 90 (7.3%) followed by Editorials 41 (3.3%), rest details are given in Table-II.

**Table-II:** Different types of manuscript published in different journals.

Type of Manuscript	Frequency (Percentage) n (%)
Original Article	1039 (84.1)
Case Report	90 (7.3)
Editorial	41 (3.3)
Review Article	23 (1.9)
Short Communication	13 (1.1)
Case Series	7 (0.6)
Letter to Editor	7 (0.6)
Pictorial	6 (0.5)
View Point	3 (0.2)
Commentary	4 (0.3)
Special Article	1 (0.1)
Study Protocol	1 (0.1)

**Table-III:** Mean difference between number of authors, journals and self citation in year 2018 and 2019.

Category	Year of publication	Mean±SD	95% Confidence Interval		P-value

			Lower limit	Upper limit	
Number of Authors	2018	4.35±1.73	-.536	-.113	0.003
	2019	4.68±2.04			
Self-citation by the Authors	2018	0.28±1.14	-.197	.066	0.33
	2019	0.35±1.22			
Self-citation by the Journal	2018	0.10±0.4	-.056	.039	0.713
	2019	0.11±0.45			
Number of references	2018	21.14±9.45	-.83	1.36	0.638
	2019	20.87±10.22			

A total of 5,587 authors were involved in all the articles published in these two years with an average of 4.52 per article. The year wise distribution of authors show that 2677 authors contributed in 2018 (average = 4.35); while in 2019, total of 2,910 authors contributed with an average of 4.69. In home authorships data, which means manuscript with a single author from the same institute as the journal is being published, showed that 502 (40.6%) of the manuscripts published had home authorship.

The study analyzed 25,893 references with an average of 20.96 references per article for authors and journal self-citations. The data revealed 390 (1.51%) references with authors’ self-citation in 139 (11.26%) manuscripts. Whereas, in journal self-citation, a total of 126 (0.49%) references in 88 (7.13%) manuscripts were recorded.

## DISCUSSION

Inappropriate authors’ and journals’ self-citation is a practice that needs to be curbed. Excessive author and journal promotion through such means also need to be discouraged.^10^ The percentage of same institute authorship detected in the present study was 40.6%, probably because it’s easier to get publication accepted in one’s home institute using references and professional influence. This practice becomes unethical when quality is overlooked during the scrutiny process.

The percentage of author self-citation came out to be 11.26%. A research conducted on three high-impact general medical journals (JAMA, Lancet, and New England Journal of Medicine) showed that self-citation was 6.5%.^3^ The reason for lower self-citation could possibly be the strict policies of these journals and the lack of such policies by the local journals of Khyber Pakhtunkhwa. A research, on diabetes-related journals, depicted that author self-citation was 18%.^1^ The trend of self-citation can be regarded as a hurdle in the process of scientific discovery if done inappropriately. It may lead to creating false links in science and giving undue credit to a research/ researcher. It can falsely inflate a researcher’s h-index and after the introduction of s-index, this practice has been looked down upon.^11^ One of the solutions for unjustified self citation practices is international collaboration. As according to Haq the most cited papers in Pakistan Library & Information Science. Journal from 2004 and 2020 had international authors and data sources.^12^

Journal self-citation came out to be 0.49%. The reason for such a low percentage could be the lack of awareness of the authors as well as the editorial board member of the local journals of KPK regarding some of the merits of self-citation. This practice of journal self citation is seen in most of the high-impact journals. This not only increases their visibility and bibliometric metrics but also their impact factor. Another trend seen is that the more an article self cites the articles in the journal it is printed in, the more citations it receives.^13^,^14^ Local literature is scant on the present topic and according to Haq in 2019 the trend of author self citation is very low in Pakistan Library and Information Science Journals. The maximum trend of citation was noted in Journal of Ayub Medical College and he was of the view that self citation trend should be promoted in Pakistan.^15^ Haq et al. evaluated the publications output of Pakistan Journal of Information Management and Libraries based on the Scopus Database and found self citation trend to be 3.68%.^16^ Livas et al checked self-citation trends in orthodontics journals and found it to be 5.71% which is fewer than many other health journals.^17^ A study on plastic and reconstructive surgery journals found their self-cited rate to be 25.8%, which was higher than other surgical specialty journals.^18^ So basically, self-citation can vary from field to field and also depends on journal type, Impact factor, and even the journal’s author specifications.^19^

The present research also shows that the number of authors increased significantly from 2018 (4.35±1.73) to 2019 (4.68±2.04). The increased number of co-authors shows higher scientific collaboration on one hand, yet, it is also an indicator of the increased number of gift authorships.^20^ According to Papatheodorou et al., the number of authors has increased by 0.8 per decade (P< 0.001). The reason for such a trend could be the growth in academia and the introduction of more complex topics.^21^

### Limitations:

Limitation of this research is that it only addresses a part of the self-citation process. We checked the synchronous type of self-citation. We did not check the citation metrics of each manuscript and journal. Moreover, self-citation was checked without assessing context. The solution can be to develop policies to curb this menace and also to use bibliometric indices.

## CONCLUSION

The study concluded substantially high number of self-citation frequency among authors, while journals show considerably low number of self-citations. The study further concluded that a high number of home authorship in various journals of Khyber Pakhtunkhwa exists.

### Authors Contribution

**MSA:** Conceived and designed the study, Data collection, final analysis, interpretation and responsible and accountable for the accuracy and integrity of the work.

**UZ:** Data collection, drafting the manuscript, paper write-up, interpretation, responsible and accountable for the accuracy or integrity of the work.

**HZ, MSU, FAK:** Data collection, drafting the manuscript, final approval of manuscript to be published.
